# Temporomandibular Disorder in Domestic Waste Collectors: Cross‐Sectional Study

**DOI:** 10.1111/joor.13975

**Published:** 2025-05-14

**Authors:** Patricia Ramos Cury, Nara Santos Araujo, Marcel Jhonnata Ferreira Carvalho, Mariana Carvalho Andrade, Daniel Araki Ribeiro, Jean Nunes dos Santos

**Affiliations:** ^1^ Department of Periodontics School of Dentistry, Federal University of Bahia Salvador Brazil; ^2^ Post‐Graduate Program in Dentistry and Health School of Dentistry, Federal University of Bahia Salvador Brazil; ^3^ Biosciences Department Federal University of São Paulo São Paulo Brazil; ^4^ Department of Oral Pathology School of Dentistry, Federal University of Bahia Salvador Brazil

**Keywords:** cross‐sectional studies, epidemiology, occupational health, temporomandibular disorder, waste workers

## Abstract

**Background:**

Musculoskeletal disorders are prevalent among waste collectors, yet the temporomandibular disorder (TMD) assessment within this group remains overlooked.

**Objective:**

This cross‐sectional study examined TMD in domestic waste collectors and its association with their work.

**Methods:**

The study involved 288 adult men from a waste collection corporation, with 130 working in domestic solid waste collection (operational workers) and 158 in other roles (controls). TMD severity was assessed through inquiries about signs and symptoms. Missing teeth were clinically evaluated. Socio‐demographic data, employment history, psychosocial factors, and severity of TMD were collected using a questionnaire.

**Results:**

TMD was diagnosed in 47% of the operational workers, and the most reported TMD factors were self‐perception of being a tense person (52.3%) and parafunctional habits (40.0%). In the whole sample, moderate/severe TMD was associated with alcohol dependence (OR = 3.51, 95% CI = 1.10–11.25, *p* = 0.03), and mild TMD was associated with parafunctional habits (OR = 3.36, 95% CI = 1.99–5.67, *p* < 0.001) and psychosocial factors (OR = 3.68, 95% CI = 2.17–6.25, *p* < 0.001). In operational workers, moderate/severe TMD was associated with alcohol dependence (OR = 4.84, 95% CI = 1.00–23.81, *p* = 0.05), and mild TMD was associated with age (OR = 2.60, 95% CI = 1.02–6.70, *p* = 0.05), psychosocial (OR = 4.41, 95% CI = 1.76–11.05, *p* = 0.002) and parafunctional (OR = 5.14, 95% CI = 2.17–12.19, *p* < 0.001) factors.

**Conclusion:**

In this sample of domestic waste collectors, moderate to severe TMD correlated with alcohol dependence, whereas mild TMD showed associations with age, psychosocial factors, and parafunctional habits. However, TMD did not exhibit a direct association with domestic waste‐collecting work. Thus, addressing physical and mental health concerns within this occupational cohort may enhance overall well‐being.

## Graphical abstract

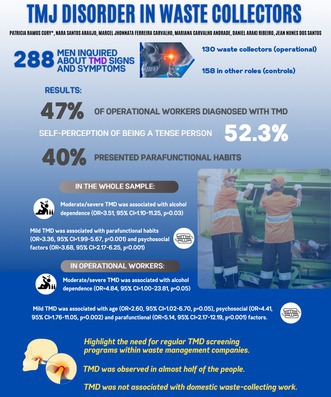

## 1 Introduction

The global municipal solid waste generated is approximately 2 billion tonnes per year, and more than 3.40 billion tonnes of municipal solid waste is expected to be generated by 2050 [1]. In this scenario, waste collection workers have a fundamental role, especially in underdeveloped countries where waste collection is performed manually. Their work is a physically demanding task associated with multiple occupational and musculoskeletal disorders. Frequently lifting heavy loads, highly repetitive tasks, long work duration, and insufficient recovery may result in chronic injuries and diseases [2, 3, 4].

Temporomandibular disorders (TMD) are a heterogeneous group of musculoskeletal and neuromuscular conditions involving the temporomandibular joint complex and surrounding musculature and skeletal components. TMD affects up to 15% of adults, with a peak incidence at 20 to 40 years. Common symptoms include jaw pain or dysfunction, earache, headache, facial pain, presence of sounds during mandibular movement, and limitation and deviation of mandibular movement [5]. The aetiology of TMD, although not completely understood, is considered multifactorial. Biomechanical, bio‐psychosocial, and biological factors may contribute to the disorder. Occlusal overloading and parafunctions (bruxism) are frequently involved as biomechanical factors. Among bio‐psychosocial factors, stress, anxiety, or depression were often encountered. Abusive consumption of psychoactive substances, including nicotine and alcohol, has also been associated with TMD [6, 7, 8]. Given the physically demanding nature of domestic waste collection, workers in this field may experience significant biomechanical stress and psychological pressure [2, 3, 4], potentially increasing their risk for TMD.

TMD can lead to personal suffering due to pain, disability, impaired quality of work and life, and a heavy socio‐economic burden for subjects [9], and has become a significant health problem among different classes of workers [6]. Although musculoskeletal disorders have been extensively documented in waste collectors, showing a prevalence of up to 85% [10, 11, 12, 13, 14], TMD has not been investigated. In the present study region (Salvador, Bahia, Brazil), garbage collection is done on a manual basis and then manually loaded into trunks. Therefore, this cross‐sectional study aimed to assess TMD among domestic waste collectors and to investigate the association between TMD and the activity of domestic waste collection. It was hypothesised that TMD is highly prevalent among collectors and is associated with waste collector activity.

## 2 Material and Methods

### 2.1 Ethical Issues

The study was conducted following the World Medical Association Declaration of Helsinki. It was approved by the Ethics and Research Committee of the School of Dentistry of the Federal University of Bahia (UFBA), Brazil (protocol 1.023.054). All participants aged > 18 received detailed explanations regarding the study and signed a consent form.

### 2.2 Study Design and Sampling Procedures

This cross‐sectional study was performed in a convenience population of 300 consecutively screened adult male individuals registered as workers at a municipal waste management company (Revita Engenharia Sustentável, Salvador, Brazil). This company is responsible for the routine collection and transport of domestic waste from private homes to disposal sites.

Waste collector activity was the exposure factor, and the TDM was the outcome. All recruited subjects worked 40 h a week. Employees with waste collection activity (operational group) gathered and transferred the waste from the residential neighbourhoods to the collection vehicle. Waste collectors engaged in physically demanding tasks, including repetitive bending, lifting, and transferring waste to collection vehicles during 8‐h shifts, five days a week. These activities were combined with prolonged standing and walking through residential neighbourhoods. The non‐exposed individuals (non‐operational group) worked as mechanics, technicians, and managers. Socio‐demographic data (age, income and education level), occupational factors (employment time and work shift), health status, and behavioural factors (smoking and drinking habits) were considered independent variables.

The inclusion criteria were male sex, age ≥ 18 years, and at least six months of employment time. The exclusion criteria were a history of neuromuscular disease. Women were excluded from the study because they represented a small proportion of workers.

### 2.3 Data Collection

A convenience sample of 288 participants was recruited between September 2016 and March 2017 at the Revita Engenharia Sustentável Company headquarters. All employees were invited to participate in the study, but only 301 were accepted; of these, 288 met all inclusion criteria. Initially, in‐person interviews were conducted by a trained researcher using a structured form to collect the following data: age, education level, monthly income, position held, employment time, shift work, medical history (any current or previous disease and current medication use, including self‐reported stress, anxiety or depression condition and medications), smoking habits, and alcohol dependence. In addition, missing teeth were examined, as previously described [15].

TMD was diagnosed based on signs and symptoms using a previously described index [16]. The index consisted of 10 items, which were asked of the individuals, as follows: I1—difficulty opening mouth wide; I2—difficulty moving jaw from side to side; I3—fatigue or muscle pain when chewing; I4—frequent headaches; I5—neck pain or wryneck; I6—earaches or pain in temporomandibular joints (TMJs); I7—clicking in the TMJs while chewing or opening mouth; I8—habit of clenching or grinding teeth; I9—teeth not articulating well; I10—tense (nervous) person according to self‐perception. For each item, there were three response options: “Yes” (scored as 10 points), “sometimes” (5 points), and “no” (0 points). The sum of the points for all items gave the overall score and allowed the following classifications: absence of TMD (0–15 points), mild TMD (20–45 points), moderate TMD (50–65), and severe TMD (70–100 points). One of the researchers instructed the volunteers to fill out the questionnaire. Each volunteer answered the questionnaire independently in a well‐lit, climate‐controlled room without time constraints.

### 2.4 Data Analysis

The statistical analysis included 288 participants (130 men who collected domestic solid waste and 158 who did not). Missing data were omitted from the analysis.

The outcome (TMD) was analysed in two ways: absent versus mild, and absent plus mild versus moderate plus severe. Age was categorised as ≤ 37 (18–37) years or > 37 years (38–61), according to the median. Education level was categorised as ≥ 9 years of education (i.e., participants who completed elementary and middle school) and < 9 years of education (i.e., participants who had not completed elementary or middle school) [17]. Economic status was categorised as monthly income ≤ US$250.00 and monthly income > US$250.00 (US$250.00 was equivalent to the Brazilian minimum wage). According to the median, employment time was categorised as ≤ 4 years or > 4 years. The work shift was categorised as either daytime or nighttime. Participants were classified as either alcohol‐dependent (based on an AUDIT test for alcohol use disorder score ≥ 8) or as alcohol nondependent [18], as current smokers or non‐smokers, and as having psychosocial factors or not. The number of missing teeth was categorised as < 5 or ≥ 5 [8], and the parafunctional habits were classified as present or absent according to self‐reported habits of clenching or grinding teeth.

A descriptive analysis was performed, calculating the absolute frequency for the categorical variables. The Fisher's exact or Chi‐squared test was used for the total sample and the operational group to assess the association between TMD (dependent variable) and the independent variable (position held). Age, education, income level, alcohol and nicotine dependence, psychosocial factors, employment time, work shift and missing teeth were also analysed as independent variables. Moreover, a stepwise logistic regression was used, adjusting for the covariates that showed *p* ≤ 0.20 in the bivariate model. The odds ratios (OR) and 95% confidence intervals (CI) were calculated.

To investigate the relationship between DTM severity and AUDIT score categories, we performed an ordinal logistic regression. DTM was categorised into three ordinal levels: absent, mild, and moderate + severe. Alcohol consumption, assessed using the AUDIT, was divided into four groups based on established risk levels: 0–7 (low risk), 8–15 (moderate risk), 16–19 (high risk), and 20–40 (probable alcohol dependence). In the model, AUDIT score categories served as the predictor variable, while DTM severity was the outcome variable. The model's fit was evaluated through the likelihood ratio chi‐square test, pseudo‐R‐squared statistics, and examination of parameter estimates and their associated *p*‐values.

*p*‐values < 0.05 were considered statistically significant. Data were analysed using a statistical software program (SPSS version 25.0, SPSS Inc., Chicago, IL, USA).

## 3 Results

Table 1 presents the characteristics of all participants based on their job positions. The majority of operational workers were aged ≤ 37 years (70%), had ≥ 9 years of education (63.1%), and had been employed for ≤ 4 years (60%), working during the day (70%). Additionally, about half of them reported experiencing psychosocial factors (52.3%) and alcohol dependence (52%). Forty percent reported having parafunctional habits, and nearly half of those individuals (47%) experienced TMD. The most reported TMD factors were self‐perception of being a tense person (52.3%) and the habit of clenching or grinding teeth (40.0%) (Table 2).

**TABLE 1:** General characteristics of the study participants (*N* = 288).

Variables	Position held		
	Total	Non‐operational worker	Operational worker
	*N* (%)		
*Socio‐demographic factors*			
Age (years)			
≤ 37	147 (51.0)	56 (35.4)	91 (70.0)
> 37	141 (49.0)	102 (64.6)	39 (30.0)
Education level (years)			
< 9	93 (32.3)	45 (28.5)	48 (36.9)
≥ 9	195 (67.7)	113 (71.5)	82 (63.1)
Monthly income (US$)			
≤ 250.00	159 (55.2)	80 (50.6)	79 (60.8)
> 250.00	129 (44.8)	78 (49.4)	51 (39.2)
*Occupational factors*			
Employment time (years)			
≤ 4	140 (48.6)	62 (39.2)	78 (60.0)
> 4	148 (51.4)	96 (60.8)	52 (40.0)
Work shift			
Day shift	225 (78.1)	134 (84.8)	91 (70.0)
Night shift	63 (21.9)	24 (15.2)	39 (30.0)
*Psychosocial factors*			
No	146 (50.7)	84 (53.2)	62 (47.7)
Yes	142 (49.3)	74 (46.8)	68 (52.3)
*Environmental factors*			
Alcohol dependence			
No	154 (53.5)	92 (58.2)	62 (47.7)
Yes	134 (46.5)	66 (41.8)	68 (52.3)
Smoking habits			
No	240 (83.3)	142 (89.9)	98 (75.4)
Yes	48 (16.7)	16 (10.1)	32 (24.6)
*Oral factor*			
Parafunctional habits			
No	190 (66.0)	112 (70.9)	78 (60.0)
Yes	98 (34.0)	46 (29.1)	52 (40.0)
Missing teeth			
< 5	197 (68.4)	97 (61.4)	100 (76.9)
≥ 5	91 (31.6)	61 (38.6)	30 (23.1)
TMD			
Absent	152 (52.8%)	83 (52.5%)	69 (53.1%)
Mild	120 (41.7%)	70 (44.3%)	50 (38.5%)
Moderate	12 (4.2%)	4 (2.5%)	8 (6.2%)
Severe	4 (1.4%)	1 (0.6%)	3 (2.3%)

**TABLE 2:** Description of the occurrence of TMD symptoms in operational workers (*N* = 130).

Factors	No	Sometimes	Yes
Difficulty opening mouth wide	121 (93.1)	3 (2.3)	6 (4.6)
Difficulty moving jaw from side to side	120 (92.3)	2 (1.5)	8 (6.2)
Fatigue or muscle pain when chewing	105 (80.8)	13 (10.0)	12 (9.2)
Frequent headaches	99 (76.2)	20 (15.4)	11 (8.4)
Neck pain or wryneck	93 (71.5)	20 (15.4)	17 (13.1)
Earaches or pain in TMJs	118 (90.8)	5 (3.8)	7 (5.4)
Clicking in TMJs while chewing or opening the mouth	108 (83.0)	11 (8.5)	11 (8.5)
Habit of clenching or grinding teeth	78 (60.0)	14 (10.8)	38 (29.2)
Teeth do not articulate well	102 (78.5)	5 (3.8)	23 (17.7)
Tense (nervous) person according to self‐perception	62 (47.7)	31 (23.8)	37 (28.5)

Although in the bivariate model, moderate/severe TMD in the whole sample was associated with operational work (Table 3), after adjusting for covariates, it was only associated with alcohol dependence (OR = 3.51, 95% CI = 1.10–11.25, *p* = 0.03). Alcohol dependence increased the chance of TMD by 3.51 (Table 4). However, ordinal logistic regression analysis revealed no significant association between AUDIT score categories and DTM severity (likelihood ratio *χ* ^2^ = 5.65, *p* = 0.13). The model explained a small proportion of the variance in DTM severity (Nagelkerke *R* ^2^ = 0.02). Although there was a marginally significant difference in the odds of having mild DTM compared to absent DTM (*p* = 0.01), none of the AUDIT categories demonstrated a statistically significant effect on DTM severity. Furthermore, mild TMD was associated with parafunctional habits (OR = 3.36, 95% CI = 1.99–5.67, *p* < 0.001) and psychosocial factors (OR = 3.68, 95% CI = 2.17–6.25, *p* < 0.001). Parafunctional and psychosocial factors increased nearly 3 times the chance of mild TMD.

**TABLE 3:** Bivariate analysis of the association between TMD (Absent/mild × Moderate/severe and Absent × Mild) and independent variables in all participants (*N* = 288).

Variable	Total	Absent/Mild	Moderate/Severe	OR (95% CI), *p*	Total	Absent	Mild	OR (95% CI), *p*
*Socio‐demographic factors*								
Age (years)				0.32 (0.10–1.04), 0.04				1.28 (0.79–2.08), 0.32
≤ 37	147 (51.0)	135 (49.6)	12 (75.0)		135 (49.6)	83 (52.2)	52 (46.0)	
> 37	141 (49.0)	137 (50.4)	4 (25.0)		137 (50.4)	76 (47.8)	61 (54.0)	
Education level (years)				2.20 (0.80–6.06), 0.12				0.91 (0543–1.54), 0.73
< 9	93 (32.3)	85 (31.3)	8 (50.0)		85 (31.3)	51 (32.1)	34 (30.1)	
≥ 9	195 (67.7)	187 (68.8)	8 (50.0)		187 (68.8)	108 (67.9)	79 (69.9)	
Monthly income (US$)				1.05 (0.38–2.89), 0.93				1.11 (0.68–1.80), 0.68
≤ 250.00	159 (55.2)	150 (55.1)	9 (56.2)		150 (55.1)	86 (54.1)	64 (56.6)	
> 250.00	129 (44.8)	122 (44.9)	7 (43.8)		122 (44.9)	73 (45.9)	49 (43.4)	
*Occupational factors*								
Employment time (years)				0.72 (0.26–2.00), 0.53				0.97 (0.58–1.57), 0.89
≤ 4	140 (48.6)	131 (48.2)	9 (56.2)		131 (48.2)	76 (47.8)	55 (48.7)	
> 4	148 (51.4)	141 (51.8)	7 (43.8)		141 (51.8)	83 (52.62)	58 (51.3)	
Work shift				3.00 (1.07–8.41), 0.03				0.74 (0.40–1.35), 0.32
Day shift	225 (78.1)	216 (79.4)	9 (56.2)		216 (79.4)	123 (77.4)	93 (82.3)	
Night shift	63 (21.9)	56 (20.6)	7 (43.8)		56 (20.6)	36 (22.6)	20 (17.7)	
Position held				2.83 (0.96–8.36), 0.05				0.86 (0.53–1.40), 0.55
Non‐operational	158 (54.9)	153 (56.3)	5 (31.2)		153 (56.3)	87 (54.7)	66 (58.4)	
Operational	130 (45.1)	119 (43.8)	11 (68.8)		119 (43.8)	72 (45.3)	47 (41.6)	
*Psychosocial factors*				1.06 (0.39–2.90), 0.91				4.10 (2.45–6.96), < 0.001
No	146 (50.7)	138 (50.7)	8 (50.0)		138 (50.7)	103 (64.8)	35 (31.0)	
Yes	142 (49.3)	134 (49.3)	8 (50.0)		134 (49.3)	56 (35.2)	78 (69.0)	
*Environmental factors*								
Alcohol dependence				3.69 (1.16–11.73), 0.02				1.15 (0.71–1.87), 0.57
No	154 (53.5)	150 (55.1)	4 (25.0)		150 (55.1)	90 (56.6)	60 (53.1)	
Yes	134 (46.5)	122 (44.9)	12 (75.0)		122 (44.9)	69 (43.4)	53 (46.9)	
Smoking habits				2.42 (0.80–7.32), 0.11				1.14 (0.59–2.19), 0.70
No	240 (83.3)	229 (84.2)	11 (68.8)		229 (84.2)	135 (84.9)	94 (83.2)	
Yes	48 (16.7)	43 (15.8)	5 (31.2)		43 (15.8)	24 (15.1)	19 (16.8)	
*Oral factors*								
Parafunctional habits				1.17 (0.41–3.33), 0.76				3.36 (1.99–5.67), < 0.001
No	190 (66.0)	180 (66.2)	10 (62.5)		180 (66.2)	123 (77.4)	57 (50.4)	
Yes	98 (34.0)	92 (33.8)	6 (37.5)		92 (33.8)	36 (22.6)	56 (49.6)	
Missing teeth				0.50 (0.17–1.48), 0.20				1.11 (0.66–1.85), 0.71
< 5	197 (68.4)	184 (67.6)	13 (81.3)		184 (67.6)	109 (68.6)	75 (66.4)	
≥ 5	91 (31.6)	88 (32.4)	3 (18.7)		88 (32.4)	50 (31.4)	38 (33.6)	

**TABLE 4:** Multiple logistic regression analysis of the relationship between the TMD and independent variables in all study participants and operational workers.

Variable	Odds ratio (95% CI)	*p*
*All sample*		
Moderate/severe TMD^a^		
Alcohol dependence	3.51 (1.10–11.25)	0.03
Mild TMD^b^		
Psychosocial factors	3.68 (2.17–6.25)	< 0.001
Parafuncional habits	3.36 (1.99–5.67)	< 0.001
*Operational workers*		
Moderate/severe TMD^c^		
Alcohol dependence	4.84 (1.00–23.81)	0.05
Mild TMD^d^		
Age	2.60 (1.02–6.70)	0.05
Psychosocial factors	4.41 (1.76–11.05)	0.002
Parafunctional habits	5.14 (2.17–12.19)	< 0.001

^**a**^ Age, educational level, missing teeth, smoking habit, alcohol dependence, position held, working shift, missing teeth.

^b^ Psychosocial factors, parafunctional habits.

^c^ Age, smoking habit, alcohol dependence, working shift.

^d^ Age, psychosocial factors, parafunctional habits.

Table 4 shows the association between TMD and putative risk factors in operational workers. After adjustment for covariates, moderate/severe TMD was significantly associated with alcohol dependence (OR = 4.84, 95% CI = 1.00–23.81, *p* = 0.05). Alcohol dependence increased by 4.84 times the chance of TMD. Mild TMD was associated with age (OR = 2.60, 95% CI = 1.02–6.70, *p* = 0.05), psychosocial (OR = 4.41, 95% CI = 1.76–11.05, *p* = 0.002), and parafunctional factors (OR = 5.14, 95% CI = 2.17–12.19, *p* < 0.001), which increased 2.6, 4.41, and 5.14 times the chance of TMD, respectively (Table 5). The bivariate analysis revealed that temporomandibular disorders (TMD) in operational workers were significantly associated with alcohol dependence (OR; 4.58; 95% CI: 1.95–10.68; *p* = 0.02), age ≥ 37 years (OR: 2.22; 95% CI: 1.01–4.88; *p* = 0.05), the presence of psychosocial factors (OR: 4.33; 95% CI: 1.93–9.70; *p* < 0.001), and parafunctional habits (OR: 6.27; 95% CI: 2.78–14.12; *p* < 0.001).

**TABLE 5:** Bivariate analysis of the association between TMD and putative risk factors in operational workers in Brazil (*N* = 128).

Variable	Total	Absent/Mild	Moderate/Severe	OR (95% CI), *p*	Total	Absent	Mild	OR (95% CI), *p*
	*N* (%)				*N* (%)			
*Socio‐demographic factors*								
Age (years)				0.21 (0.03–1.73), 0.10				2.22 (1.01–4.88), 0.05
≤ 37	91 (70.0)	81 (68.1)	10 (90.9)		81 (68.1)	54 (75.0)	27 (57.4)	
> 37	39 (30.0)	38 (31.9)	1 (9.1)		38 (31.9)	18 (25.0)	20 (42.6)	
Education level (years)				1.47 (0.42–5.11), 0.38				0.86 (0.40–1.86), 0.70
< 9	48 (36.9)	43 (36.1)	5 (45.5)		43 (36.1)	27 (37.5)	16 (34.0)	
≥ 9	82 (63.1)	76 (63.9)	6 (54.5)		76 (63.9)	45 (62.5)	31 (66.0)	
Monthly income (US$)				1.14 (0.32–4.12), 0.56				0.70 (0.33–1.48), 0.35
≤ 250.00	79 (60.8)	72 (60.5)	7 (41.2)		72 (60.5)	46 (63.9)	26 (55.3)	
> 250.00	51 (39.2)	47 (39.5)	4 (36.4)		47 (39.5)	26 (36.1)	21 (44.7)	
*Occupational factors*								1.60 (0.76–3.36), 0.22
Employment time (years)				0.31 (0.06–1.48), 0.11	69 (58.0)	45 (62.5)	24 (51.1)	
≤ 4	78 (60.0)	69 (58.0)	9 (81.8)		50 (42.0)	27 (37.5)	23 (48.9)	
> 4	52 (40.0)	50 (42.0)	2 (18.2)					
Work shift				3.13 (0.89–10.95), 0.07				0.69 (0.30–1.61), 0.39
Day shift	91 (70.0)	86 (72.3)	5 (45.5)		86 (72.3)	50 (69.4)	36 (76.6)	
Night shift	39 (30.0)	33 (27.7)	6 (54.5)		33 (27.7)	22 (30.6)	11 (23.4)	
*Psychosocial factors*				0.49 (0.14–1.77), 0.22				4.33 (1.93–9.70), < 0.001
No	62 (47.7)	55 (46.2)	7 (63.6)		55 (46.2)	43 (59.7)	12 (25.5)	
Yes	68 (52.3)	64 (53.8)	4 (36.4)		64 (53.8)	29 (40.3)	35 (74.5)	
*Environmental factors*								
Alcohol dependence				4.58 (1.95–10.68), 0.02				0.83 (0.40–1.74), 0.63
No	62 (47.7)	60 (50.4)	2 (18.2)		60 (50.4)	35 (48.6)	25 (53.2)	
Yes	68 (52.3)	59 (49.6)	9 (81.8)		59 (49.6)	37 (51.4)	22 (46.8)	
Smoking habits				2.84 (0.80–10.03), 0.10				1.30 (0.55–3.10), 0.55
No	98 (75.4)	92 (77.3)	6 (54.5)		92 (77.3)	57 (79.2)	35 (74.5)	
Yes	32 (24.6)	27 (22.7)	5 (45.5)		27 (22.7)	15 (20.8)	12 (25.5)	
*Oral factors*								
Parafunctional habits				0.85 (0.24–3.05), 0.53				6.27 (2.78–14.12), < 0.001
No	78 (60.0)	71 (59.7)	7 (63.6)		71 (59.7)	55 (76.4)	16 (34.0)	
Yes	52 (40.0)	48 (40.3)	4 (36.4)		48 (40.3)	17 (23.6)	31 (66.0)	
Missing teeth				0.31 (0.04–2.54), 0.22				1.11 (0.47–2.60), 0.81
< 5	100 (76.9)	90 (75.6)	10 (90.9)		90 (75.6)	55 (76.4)	35 (74.5)	
> 5	30 (23.1)	29 (24.4)	1 (9.1)		29 (24.4)	17 (23.6)	12 (25.5)	

## 4 Discussion

In this cross‐sectional study, we evaluated TMD in domestic waste collectors and the association between TMD and domestic waste collection activity. TMD was observed in almost half of the people; mild TMD was associated with age, parafunctional, and psychosocial factors, whereas moderate/severe TMD was associated with alcohol dependence. However, TMD was not related to domestic waste‐collecting work. Therefore, the original hypothesis was not confirmed.

TMD occurs in approximately half of the operational and non‐operational individuals within the previously reported rates between 50% and 70% worldwide [19]. The prevalence of TMD found in different categories of workers varies widely, with 24.3% among dentists [20], 33.3% in civilian pilots [21], 39% among state police drivers [22], 42.3% among computer office workers [23], 58% among professional musicians [24] and 74.4% among nursing professionals [25]. Although data on TMD in waste collectors is not available, musculoskeletal disorders showed a high concentration of cases in waste collectors [13]. Given this, it was expected that the occupational status of the evaluated sample would play an important role in the occurrence of TMD. Interestingly, the study found no significant relationship between TMD and domestic waste‐collecting work. This suggests that occupational factors related to this type of work may not significantly impact the development or severity of TMD symptoms. One possible explanation is that the physical demands of waste‐collecting work may involve repetitive body movements that predominantly affect other musculoskeletal regions rather than the temporomandibular joint. Alternatively, the ergonomic adaptations or task‐specific routines in waste collection may not exert significant mechanical stress on the jaw and associated structures. Overall, these findings underscore the multifactorial nature of TMD, with a complex interplay of biological, behavioural, and psychosocial factors contributing to its onset and severity in different individuals.

Alcohol dependence was the only factor associated with moderate and severe TMD in the operational and the whole group, but not in the control group (Data not shown: Mild TMD, *p* = 0.37; Moderate/severe TMD, *p* = 0.13). Although the literature on the association between the consumption of alcohol and TMD is vague, which enhances the value of the present study, a cross‐sectional study of young adults who entered military service reported that the prevalence of TMD symptoms is linked with an increased frequency of alcohol consumption, and alcohol consumption once a week or more is often significantly associated with TMD symptoms [25]. In contrast, a cross‐sectional survey‐based study of 676 students in biomedical studies did not find a significant association between alcohol consumption and TMD symptoms [26]. Given the analgesic capacity of alcohol in deep pain [27], it could be suggested that alcohol consumption might have been higher in workers with TMD to alleviate the pain. Furthermore, the findings may also indicate a bidirectional relationship between alcohol use and TMD, as alcohol dependence may contribute to the development or exacerbation of TMD, and conversely, individuals with TMD may turn to alcohol to cope with their symptoms. Alcohol consumption could also serve as an alternative means of relieving daily tensions, as 52% of operational workers reported feeling tense. However, it is important to note that alcohol consumption as measured by the AUDIT was not significantly associated with DTM severity.

Mild TMD was associated with psychosocial and parafunctional factors. A tense or nervous person may experience heightened levels of anxiety, stress, and other emotional states. The psychological distress can lead to behaviours that trigger TMD, such as teeth grinding, clenching, and tooth‐contacting habits, which exert excessive stress on the temporomandibular joint and masticatory muscles, leading to pain and restricted jaw movements [28]. Furthermore, the increased psychological distress can cause vasoconstriction around the temporomandibular joint area, resulting in insufficient blood flow to the masticatory muscles. This reduces adenosine triphosphate levels necessary for muscle relaxation and lowers the pain threshold, leading to the development and worsening of TMD symptoms [29]. Thus, addressing mental health issues in this occupational group may maximise the worker's well‐being and reduce TMD.

Regarding age, we found that higher age increased 2.6 times the chance of mild TMD. Recent studies agree with this result, showing that the peak prevalence occurs in 45–64 year‐olds. Similar to other joints, the temporomandibular joint degenerates with age [30]. In most temporomandibular joint degeneration patients, the clinical symptoms are minimal [31], which may explain why only mild TMD was associated with age.

The present study revealed that psychosocial factors increased 4.41 the chance for mild TMD and that 53% of operational workers reported experiencing psychosocial factors (53%), which included stress, anxiety, or depression, with only one individual under medication for depression. The evaluated operational workers performed their activities under arduous physical and psycho‐emotional conditions, under an environment of stress for most of their work hours. A positive association between psychosocial factors and TMD across other job categories has been reported [6], and it has been considered a possible etiologic factor for TMD [32]; once stress may act as an agent of somatic hyperactivity of the masticatory muscles, triggering muscle and joint changes and consequent pain and function [33].

In the present study, mild TMD was associated with parafunctional habits, increasing 5.14 times the chance of TMD. Parafunctional habits, such as teeth grinding or clenching, have long been suggested as potential risk factors for TMD [8]. The mechanisms through which these habits contribute to TMD onset and progression may involve multifactorial processes, including the imposition of excessive and sustained mechanical stress on the masticatory muscles and articulation.

This study has a few limitations. Analysing a convenience sample in a single company and examining their workplace may lead to poor representation of thehigh‐risk population. For this reason, other waste collection companies should be studied. Performing the interview at their workplace might have excluded subjects with negativeself‐perceptions of their health, who might have refused to participate to avoid personal exposure. While a more systematic sampling strategy—rather than convenience sampling—would enhance the study's representativeness and minimise selection bias, implementing such a method would have been impractical and potentially unfeasible for this study. Nevertheless, the power analysis conducted for mild and moderate/severe TMD indicated an adequate sample size. The post hoc power calculation showed 78.8% for mild TMD and 100% for moderate/severe TMD in a one‐sided test with an alpha level of 0.05. A standardised clinical examination using the Diagnostic Criteria for Temporomandibular Disorders criteria would provide more robust diagnostic accuracy compared to the current self‐reported symptoms approach [34].

This cross‐sectional study simultaneously measured exposure and health outcomes; therefore, it is impossible to conclude the cause‐effect relationship. Thus, longitudinal studies should be planned to establish a temporal relationship between these variables. Future research should also focus on cost–benefit analyses of preventive interventions to justify workplace health programmes. Additionally, studies should investigate the effectiveness of specific interventions, such as stress management programmes or ergonomic modifications, to better understand their impact on TMD development.

The findings of this study highlight the need for regular TMD screening programs within waste management companies, emphasising both physical and psychological assessments. Healthcare providers working with this population should prioritise alcohol dependence screening. Preventive measures should integrate stress management techniques, ergonomic training to mitigate workplace‐related risks, and educational initiatives to address and reduce parafunctional habits. Additionally, treatment strategies must be tailored to the unique occupational challenges of waste collectors, considering their demanding work schedules and the physical nature of their tasks to ensure effective management and improved health outcomes. Workplace policies should also address psychosocial health and target alcohol dependence risks, creating a more supportive and healthier environment.

Implementing these findings into practice may require a phased approach, beginning with basic TMD screening integrated into routine health checks. Preventive education addressing key risk factors should follow, alongside clear referral pathways for timely intervention. Regular monitoring and evaluation are essential to refine strategies and demonstrate programme effectiveness. Collaboration between occupational health services, TMD specialists, and mental health professionals is crucial to provide a comprehensive and holistic approach to worker health.

In conclusion, TMD was observed in almost half of the people. In this sample of domestic waste collectors, moderate/severe TMD was associated with alcohol dependence, while mild TMD was related to age, psychosocial, and parafunctional factors. TMD was not associated with domestic waste‐collecting work. Thus, addressing physical and mental health issues in this occupational group may maximise their well‐being, mitigating symptomatology and enhancing workers' productivity. Future studies should include data from multiple waste management companies to improve the generalisability of the findings in this occupational group.

## Author Contributions

**Patricia Ramos Cury:** principal investigator, conceived and designed the study, supervised the research process, critically reviewed and revised the manuscript. **Nara Santos Araujo:** collected data and conducted the initial statistical analyses, contributed to the interpretation of results and drafting of the manuscript. **Marcel Jhonnata Ferreira Carvalho:** conducted data collection and participated in the development of the research protocol, contributed to manuscript writing. **Mariana Carvalho Andrade:** collected data and conducted the initial statistical analyses. **Daniel Araki Ribeiro:** provided expertise in data analysis and interpretation, contributed to manuscript revision and final approval. **Jean Nunes dos Santos:** contributed to the conceptual framework of the study, participated in manuscript drafting and critical revision. All authors read and approved the final manuscript, and all agree to be accountable for all aspects of the work, ensuring its accuracy and integrity.

## Ethics Statement

The project was approved by the Ethics and Research Committee of the School of Dentistry of the Federal University of Bahia (UFBA), Brazil (protocol 1.023.054). All participants signed a consent form.

## Conflicts of Interest

The authors declare no conflicts of interest.

## Peer Review

The peer review history for this article is available at https://www.webofscience.com/api/gateway/wos/peer‐review/10.1111/joor.13975.

## Data Availability

Data will be made available upon request.
